# Strong Uptake of Gas-Phase Organic Peroxy Radicals (ROO^•^) by Solid Surfaces Driven by Redox Reactions

**DOI:** 10.1021/jacsau.4c00060

**Published:** 2024-04-30

**Authors:** Olivier Durif, Felix Piel, Armin Wisthaler, Barbara Nozière

**Affiliations:** † Department of Chemistry, KTH Royal Institute of Technology, Stockholm 10044, Sweden; ‡ Department of Chemistry, University of Oslo, Oslo 0315, Norway

**Keywords:** peroxy radical, uptake, surface uptake, kinetics, PTRMS, redox, mass spectrometry, RO2

## Abstract

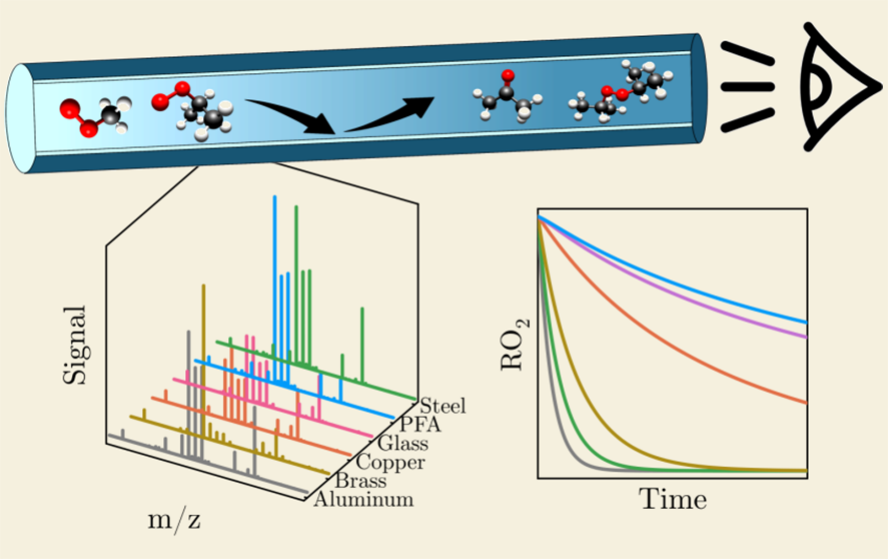

Organic peroxy radicals (ROO^•^) are key oxidants in a wide range of chemical systems such as living organisms, chemical synthesis and polymerization systems, combustion systems, the natural environment, and the Earth’s atmosphere. Although surfaces are ubiquitous in all of these systems, the interactions of organic peroxy radicals with these surfaces have not been studied until today because of a lack of adequate detection techniques. In this work, the uptake and reaction of gas-phase organic peroxy radicals (CH_3_OO^•^ and i-C_3_H_7_OO^•^) with solid surfaces was studied by monitoring each radical specifically and in real-time with mass spectrometry. Our results show that the uptake of organic peroxy radicals varies widely with the surface material. While their uptake by borosilicate glass and perfluoroalkoxy alkanes (PFA) was negligible, it was substantial with metals and even dominated over the gas-phase reactions with stainless steel and aluminum. The results also indicate that these uptakes are controlled by redox reactions at the surfaces for which the products were analyzed. Our results show that the reactions of organic peroxy radicals with metal surfaces have to be carefully considered in all the experimental investigations of these radicals as they could directly impact the kinetic and mechanistic knowledge derived from such studies.

## 1 Introduction

Organic peroxy radicals (ROO^•^) are key oxidants and reaction intermediates in a large number of natural and man-made chemical systems, such as living organisms and human health,^1−3^ chemical synthesis and polymerization,^4^ combustion,^5^ environmental chemistry,^6^ atmospheric chemistry,^7,8^ and food science,^9,10^ to name but a few. But the direct observation of these radicals remains experimentally challenging. Thus, most investigations of their chemistry are indirect, i.e., based on monitoring other compounds. For instance, in oximetry,^11^ a technique used to determine the activity of an oxidant in biochemistry or in chemical amplification,^12^ which is used to measure HOO^•^ and ROO^•^ in the atmosphere, the radicals are converted into O_2_, NO_2_, or other compounds, which are then measured. Other common techniques for the detection of organic peroxy radicals, such as electron spin resonance (ESR)^13,14^ and optical spectroscopies,^15,16^ are direct, but with a few exceptions, do not distinguish between different ROO^•^ radicals. They thus lead to some significant uncertainties in the results as, in nearly all cases, more than one radical is present in the system and contributes to the observed signals. Mass spectrometry, on the other hand, allows for the direct detection of individual organic peroxy radicals by monitoring their specific ions^17−29^ and is thus a suitable tool for monitoring these radicals in a variety of chemical systems and advancing the understanding of their complex chemistry.

In addition, surfaces are present in all of the chemical systems mentioned above: membranes in living organisms, engine or furnace walls in combustion systems, or aerosol particles in the atmosphere, among others. The organic peroxy radicals can potentially react with these surfaces in competition with their bulk reactivity, which could affect their bulk concentration. This is known to be the case, for instance, for the hydroperoxy radical (HOO^•^) in the atmosphere,^30,31^ where its reactions with aerosol surfaces can significantly affect its gas-phase concentration. Reactions with surfaces inside chemical amplification instruments are also known to be the main losses both for ROO^•^ and HOO^•^.^12^ However, because of the technical difficulties in monitoring individual organic peroxy radicals mentioned above, their interactions with surfaces have hardly been studied until today.^20,32−34^

In this work, the real-time uptake of individual gas-phase peroxy radicals by solid surfaces was monitored, and the compounds produced by these interactions were analyzed using chemical ionization mass spectrometry with proton-transfer reactions.^17,35,36^ The results show that the uptake of organic peroxy radicals varies widely with the surface material. In particular, it can be very large with some metals and compete with their gas-phase chemistry, which has direct implications on the measurement of these radicals with most detection techniques and laboratory setups. We also demonstrate that these uptakes are kinetically controlled by the redox potentials.

## 2 Results

### 2.1 Real-Time Observation of the Uptake

**Figure 1:**
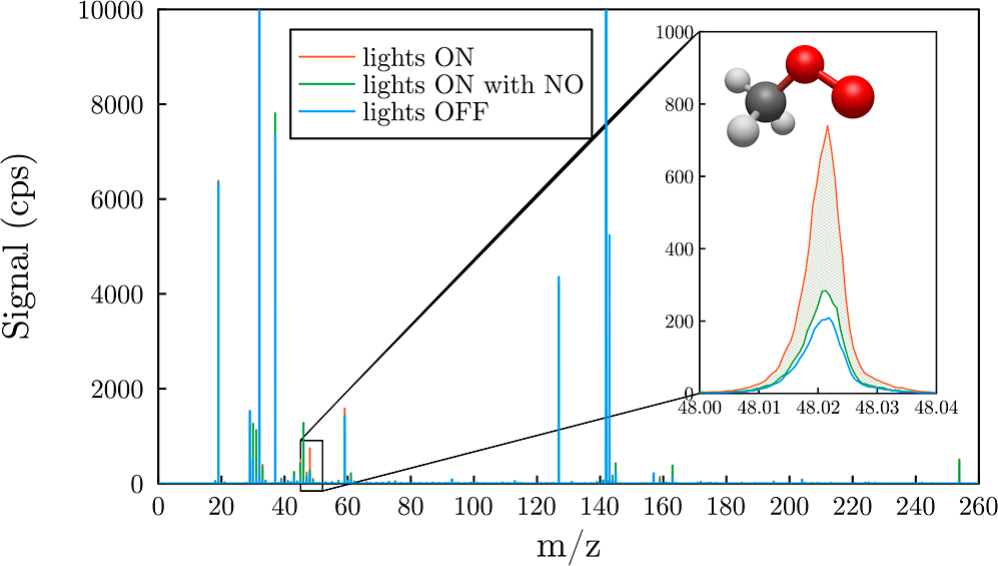
Detection of CH_3_OO^•^ by proton-transfer-reaction mass spectrometry. To ensure that the signals monitored were exclusively those of the peroxy radicals, nitric oxide was introduced (spectrum in green). As illustrated here for methylperoxy, the ROO^•^ obtained after photolysis (spectrum in orange) was subtracted from the background signal corresponding to the hashed area at *m*/*z* 48.021(3) ().

**Figure 2:**
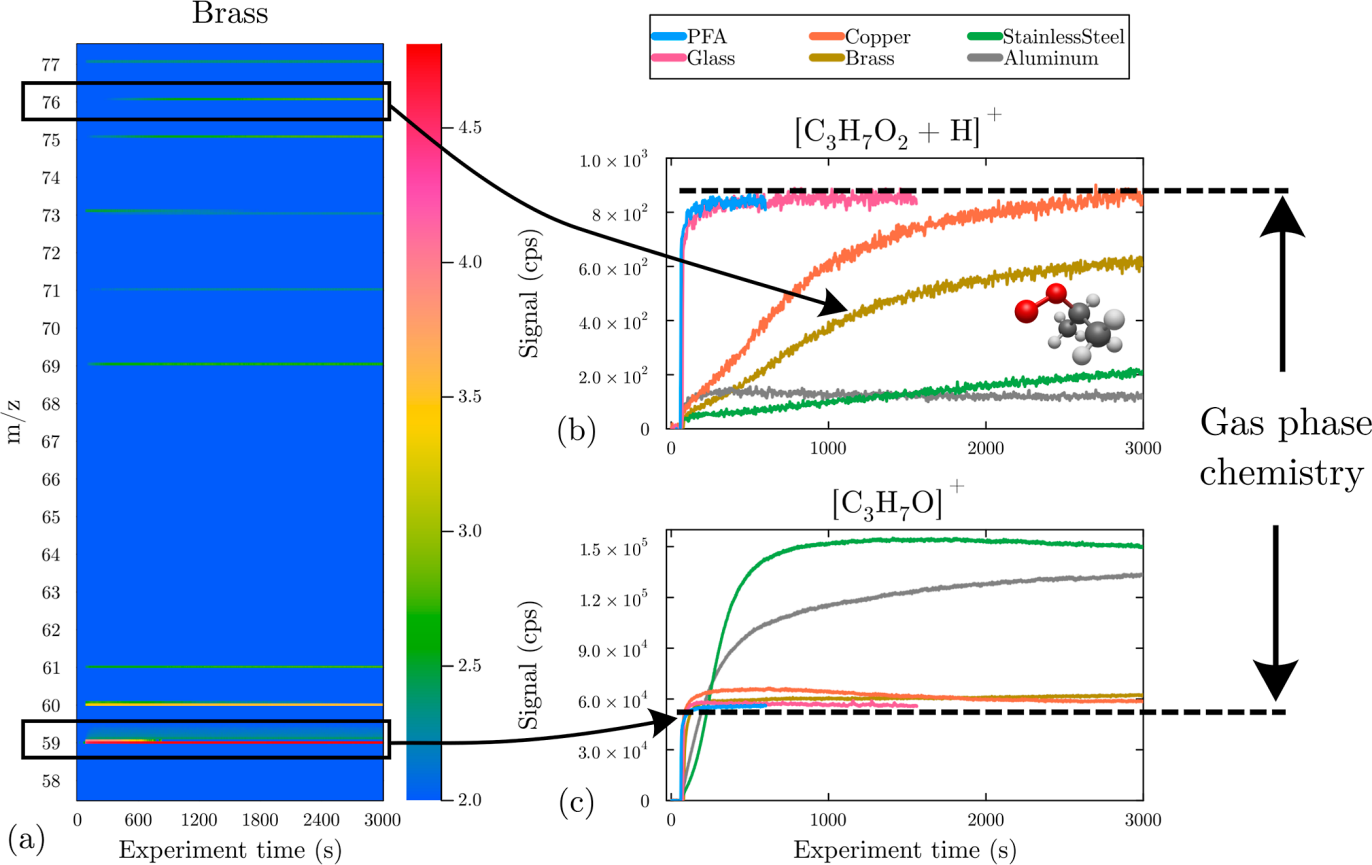
Time-dependent mass spectra, uptake, and product formation for i-C_3_H_7_OO^•^ exposed to various surfaces. (a) Time evolution of the mass spectra obtained by proton-transfer chemical ionization after photolysis in the presence of a brass surface (signal in cps in color code in the log scale as a function of ion *m*/*z* and experiment time). Time profiles (b) for the radical (*m*/*z* 76.052) and (c) main product ion (*m*/*z* 59.049), with different surface materials. The dotted lines represent the signal expected only from the gas-phase reactions.

To study the uptake of organic peroxy radicals by solid surfaces, methylperoxy (CH_3_OO^•^) and iso-propylperoxy (i-C_3_H_7_OO^•^) radicals were produced photolytically in a flow of dry, synthetic air at atmospheric pressure and flown through tubing made of different material: aluminum, 316 stainless steel, brass (10% zink), copper, borosilicate glass, and perfluoroalkoxy alkanes (PFA). A proton-transfer-reaction time-of-flight mass spectrometer (PTR-TOF-MS)^37^ placed at the exit of the surfaces was employed to monitor the radicals and their stable products in the gas (details in the Supporting Information, SI). The radicals were detected in their protonated form (). For unambiguous identification, nitric oxide (NO) was periodically introduced into the instrument subsampling flow to suppress all ROO^•^ radicals. These tests of the reliability of the ROO^•^ signal were performed intermittently, while the analyses reported in this work correspond to experiments conducted without adding NO to avoid any complex chemistry interfering with the measurements. The ROO^•^ signals were thus obtained by systematically subtracting the residual background without photolysis (Figure 1). Once photolysis was initiated, producing the radicals, mass spectra were acquired every second, while the contact time between the radical and the surface was kept constant. The radical signal as a function of the experiment time was extracted from these time-dependent spectra (Figures 2 and S2).

The time profiles observed for the radicals varied widely with the surface materials but followed the same trends for CH_3_OO^•^ and i-C_3_H_7_OO^•^. With PFA and borosilicate glass, immediately after the radicals were produced photolytically, the radical signal increased and stabilized quickly to a maximum level, which corresponded to the radical concentration resulting from gas-phase chemistry alone.

In the presence of metal surfaces, the radical signal was markedly lower than that with glass and PFA, thus indicating a significant uptake (Figures 2b and S2b). With copper and brass, the signal increased slowly to eventually reach the same level as that with glass and PFA. With stainless steel and aluminum, the signal displayed a considerable uptake, stabilizing at a low value with aluminum and showing a very slow but continuous increase over time with stainless steel (Figures 2b and S2b). These observations thus indicated that the radicals did not react on PFA and borosilicate glass surfaces but were significantly adsorbed or reacted with metals.

The reversibility of the uptake by the different surface materials was investigated by temporarily stopping the photolysis, thus the production of radicals (Figure S2b). In the absence of incoming radicals, their signal decreased quickly and did not recover, thus demonstrating that the uptake was irreversible.

### 2.2 Kinetic Analysis

**Figure 3:**
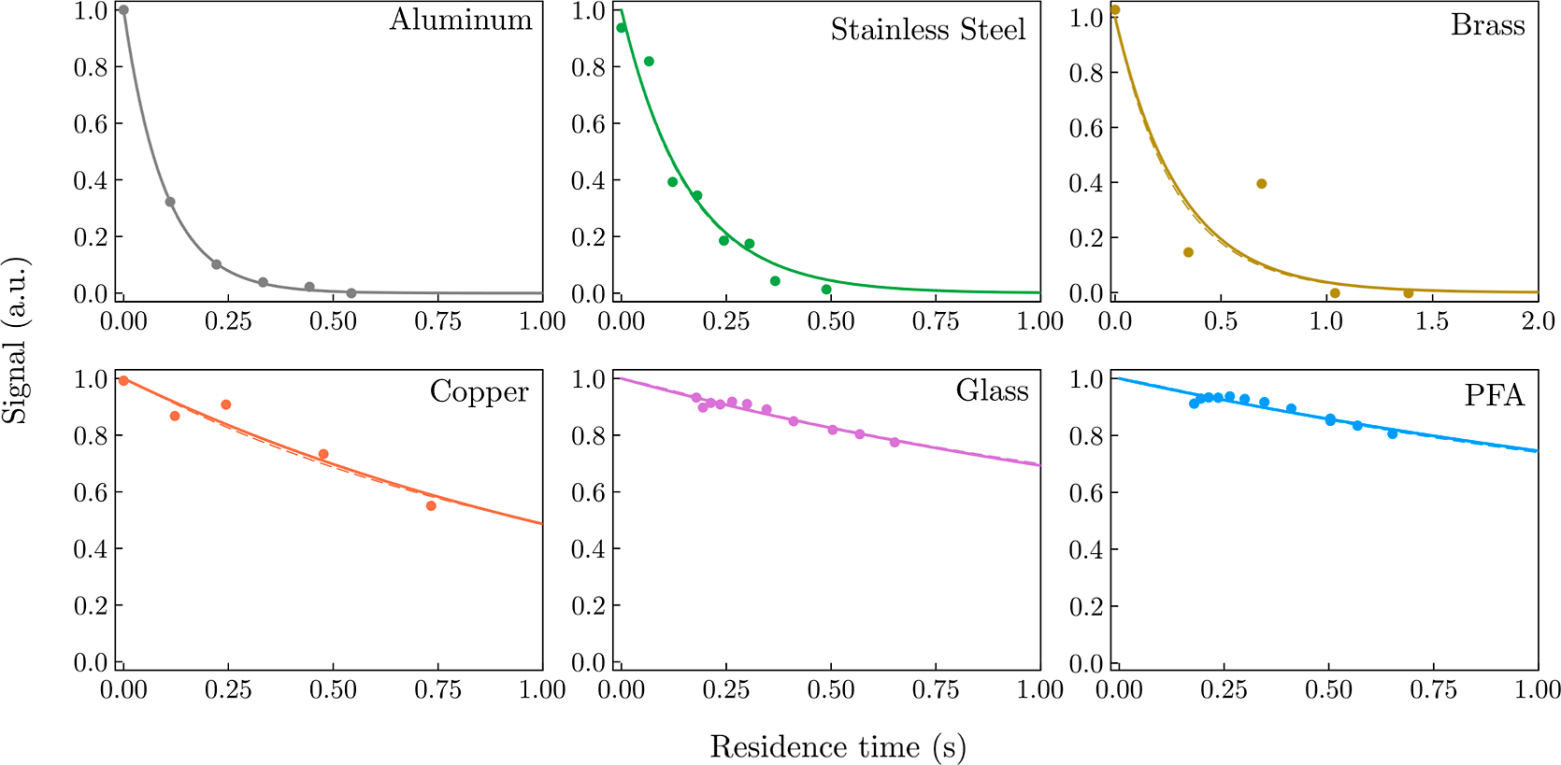
Decays of CH_3_OO^•^ as a function of residence time with different surface materials. For each residence time, the radical signal was averaged over an experiment time of 30 to 60 s following the start of photolysis. These decays were analyzed kinetically to determine the uptake coefficients. The solid lines represent the results of the analytical fit and the dashed lines represent the results of the numerical model.

To investigate these processes, the radical signals were monitored at a constant and short experiment time, while the contact time between the radicals and the surfaces was varied (hereafter termed “residence time” and corresponding to a surface area exposed to a given flow of radicals). The decays obtained for CH_3_OO^•^ are illustrated in Figure 3 and in Figure S3 for i-C_3_H_7_OO^•^.

The kinetic results enabled quantification of the decay rates, which provided a unimolecular rate constant for the reactions with the surfaces, *k*′, and an uptake coefficient, γ. The results are reported in Table 1.

**Table 1:** Decay Rates and Uptake Coefficients for Gas-Phase CH_3_OO^•^ and i-C_3_H_7_OO^•^ for the Different Surfaces^a^

	CH_3_OO^•^		C_3_H_7_OO^•^	
material	*k*′ (s^–1^)	γ	*k*′ (s^–1^)	γ
aluminum	10	10^–4^	4	6 × 10^–^^5^
stainless steel	6	7 × 10^–^^5^	2	3 × 10^–^^5^
brass	3	4 × 10^–^^5^	1	2 × 10^–^^5^
copper	0.5	<5 × 10^–^^6^	10^–1^	10^–6^
borosilicate glass	<8 × 10^–^^2^	<6 × 10^–^^7^	<10^–2^	<10^–7^
PFA	<10^–2^	<10^–7^	<5 × 10^–^^3^	<7 × 10^–^^8^

a The exposure time was between 30 and 60 s. The uncertainty is estimated to be 20%, both on the rate and uptake coefficients.

The kinetic results showed that, with borosilicate glass and PFA, the observed radical decays predominantly resulted from gas-phase reactions, whereas the uptake by the surface was negligible. Therefore, in Table 1, only upper limits for these uptake coefficients are provided. In contrast, the uptake by metal surfaces was substantial, and the gas-phase chemistry was negligible compared with the surface processes. The uptake coefficient was also determined after a longer experiment time. The results are presented in Table S1. Note that exposing these surfaces to high concentrations of peroxy radicals and precursors (approximately 1 ppm) over long experiment times is likely to have affected them.

The kinetic results confirmed that for both radicals, the uptake coefficients vary widely with the surface material. The kinetic decays obtained showed that the radical uptake was substantially larger with metals than that with borosilicate glass and PFA (illustrated by Figure 3 for CH_3_OO^•^ and Figure S3 for i-C_3_H_7_OO^•^). The materials exhibiting larger uptakes were also those showing more time dependence (signal continuously increasing after photolysis because of wall effects; see Figures 2b and S2b). The uptake coefficient was also systematically larger for CH_3_OO^•^ than that for i-C_3_H_7_OO^•^, scaling up with the intrinsic reactivity of the radical; i-C_3_H_7_OO^•^, being a substituted (secondary) radical, is less reactive than CH_3_OO^•^.

The uptake coefficient reported in Table 1 for metal surfaces decreased in the order aluminum > stainless steel > brass > copper, thus following the electropotential series of the metals in highly corrosive environments.^38^ This suggested that the uptake involved redox reactions that are driven by the redox potential with an electron exchange between the radical and the surface. PFA and borosilicate glass are electrical insulators and thus cannot exchange electrons with radicals or promote redox chemistry, which is consistent with the lack of uptake by these materials. Since the uptakes were irreversible, their transitional profiles (slow increase of the radical signal after photolysis, in Figures 2b and S2b) could not be explained by reversible processes such as adsorption (sticking) of the radicals. Instead, these time-dependent profiles were explained by the progressive formation of a bond between the radical and the surface material, ultimately leading to saturation of the surface. With stainless steel, brass, and copper, this progressive saturation resulted in a slow increase in the peroxy radical signal. In the specific case of aluminum, the buildup of this surface layer appeared to be nonkinetically limiting, resulting in a low and flat radical time profile (Figures 2b and S2b).

The potential effects of secondary iodine chemistry on the uptake of the peroxy radicals have been ruled out, as discussed in section 1.2 of Supporting Information.

### 2.3 Products of Surface Reactions

The occurrence of reactions between the radicals and the surfaces was further confirmed by investigating the products released in the gas upon contact between the radicals and the surfaces. For this, the compounds present in the gas phase when the different surfaces were exposed to CH_3_OO^•^, C_2_H_5_OO^•^, and i-C_3_H_7_OO^•^ were analyzed both by  and  chemical ionization. The ions detected in each case are reported in Tables 2 and 3, respectively. Note that in the following discussion, only the compounds produced in concentrations larger than those of the gas-phase reactions alone were analyzed.

**Table 2:** Main Ions as Products of the Peroxy Radical Reactions Detected by [H_3_O]^+^ Chemical Ionization^a^

detected products	CH_3_OO^•^	C_2_H_5_OO^•^	i-C_3_H_7_OO^•^
[R_–H_O + H]^+^	high signal enhanced by metals	high signal enhanced by metals	high signal enhanced by metals
[ROH + H]^+^	low signal enhanced by metals	not detected	not detected
[ROOR + H]^+^	not detected (fragmentation)	not detected (fragmentation)	low signal enhanced on copper and brass reduced on steel and alum

a [R_–H_O + H]^+^ ions correspond to the sum of R_–H_O and ROOR because of fragmentation at the ionization. The [ROH + H]^+^ ion is identified to the signal of the corresponding alcohol.

**Table 3:** Main Ions as Products of the Peroxy Radical Reactions Detected by [NH_4_]^+^ Chemical Ionization^a^

detected products	CH_3_OO^•^	C_2_H_5_OO^•^	i-C_3_H_7_OO^•^
[R_–H_O + NH_4_]^+^	not detected	high signal enhanced by metals	high signal enhanced by metals
[ROH + NH_4_]^+^	not detected	not detected	low signal enhanced by metals
[ROOR + NH_4_]^+^	not detected	low signal enhanced by metals	low signal enhanced by metals

a Ion products detected by the  adduct are attributed to their neutral counterparts. Products of CH_3_OO^•^ were not detected but likely because of the low binding energy with the  adduct.

#### 2.3.1 Product Detection by [H_3_O]^+^

With the hydronium ionization, the main ion observed for all the radicals corresponded to the *m*/*z* of their corresponding carbonyl compound, [R_–H_O + H]^+^: , , and  for CH_3_OO^•^, C_2_H_5_OO^•^, and i-C_3_H_7_OO^•^, respectively. As displayed in Figures 2c and S2c, the signals for these [R_–H_O + H]^+^ ions reached a maximum after a certain experimental time, which was higher with the metal surfaces than with PFA and borosilicate glass. With PFA and borosilicate glass, since the radicals were not significantly taken up by the surfaces, the compounds observed were assumed to result only from the gas-phase reactions of the radicals, to which reactions with the small metal surfaces in the experimental setup, such as the inlet of the instrument, were considered to have a negligible contribution. Thus, the excess signals for the ion [R_–H_O + H]^+^ obtained in the presence of metal surfaces compared to those in the presence of glass and PFA indicated that they corresponded to products formed by surface reactions and released in the gas phase.

However, previous works^39,40^ indicated that [R_–H_O + H]^+^ ions could result from different neutral parents. In addition to carbonyl products, they could also be [RO^+^] ions resulting from hydroperoxide (ROOH) or organic peroxide (ROOR) products because of fragmentation during proton transfer or energized collisions within the drift tube. This was confirmed in the present work by performing calibrations with standards of *tert*-butyl hydroperoxide [(CH_3_)_3_COOH] and *tert*-butyl–OO–*tert*-butyl [(CH_3_)_3_COOC(CH_3_)_3_], which both produced the ion [R_–H_O + H]^+^. With CH_3_OO^•^, in addition to the ion , a small production of the ion  was also observed, which corresponded to methanol (see Figure S2d). This was consistent with methanol being reported as the main product of this radical with metal nanoparticles in the aqueous phase.^41^ However, the formation of the corresponding alcohols for C_2_H_5_OO^•^ (ethanol) and i-C_3_H_7_OO^•^ (iso-propanol) was not observed in the present experiments.

Finally, for CH_3_OO^•^ with aluminum, much fewer products were observed in the gas than in the presence of its gas-phase reactions alone, despite the large uptake measured for this radical on this material. This was attributed to the strong adhesiveness of the expected products on this metal, which was consistent with the slow decay of the total product signal observed with this metal once the photolysis was stopped (Figure S2c).

#### 2.3.2 Product Detection by [NH_4_]^+^

**Figure 4:**
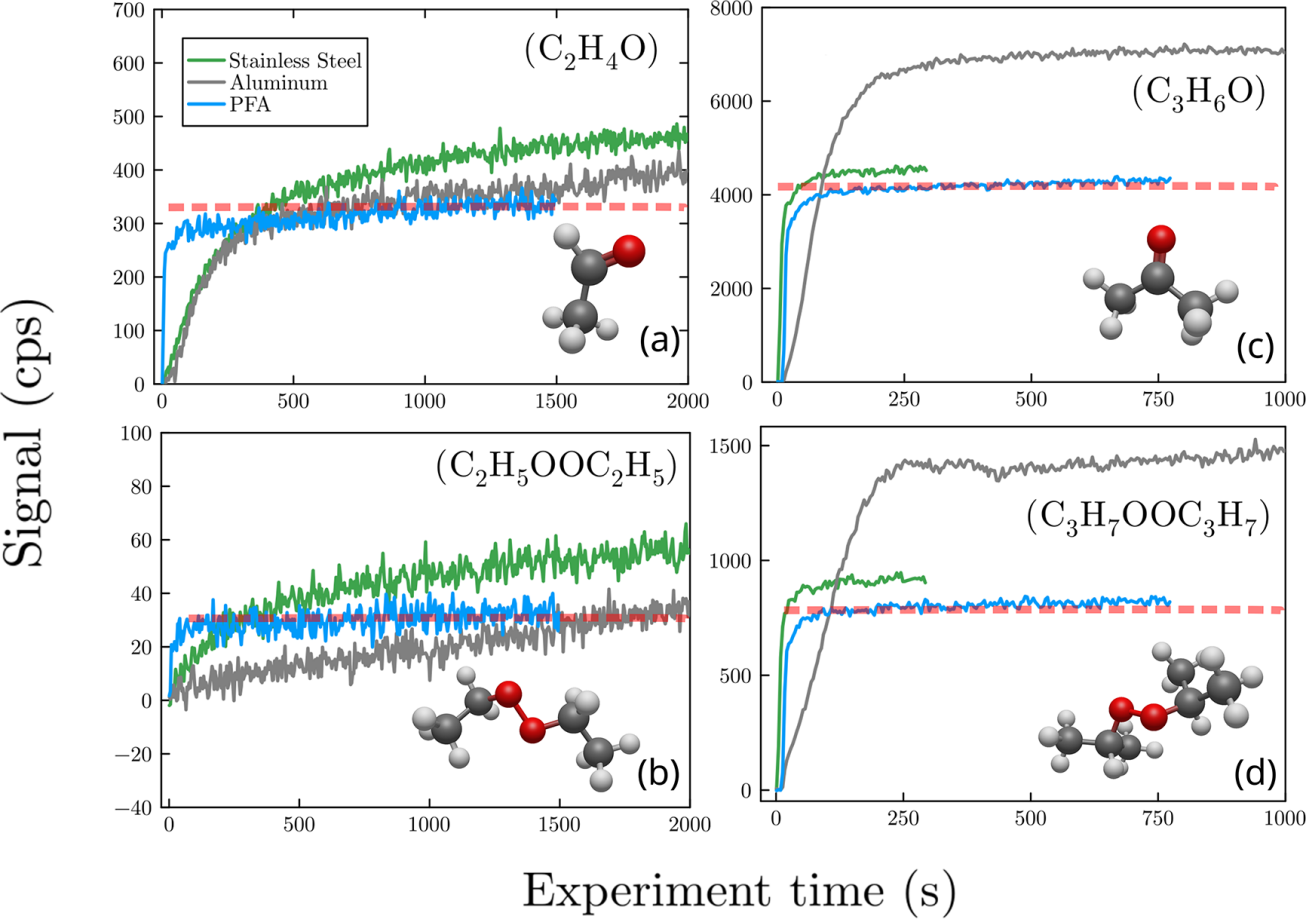
Times profile for the main ion products observed with  ionization. First column: C_2_H_5_OO^•^ products (a) *m*/*z* 62.060(6) () and (b) *m*/*z* 108.102(11) (). Second column: i-C_3_H_7_OO^•^ products (c) *m*/*z* 76.076(8) (), (d) *m*/*z* 136.133(14) (). The dashed line corresponds to the gas-phase level.

The identity of the products observed at the ions [R_–H_O + H]^+^ was further investigated by performing an analysis of the gas-phase mixture with ammonium ion adduct chemical ionization (Figure 4). Calibrations of standards of *tert*-butyl hydroperoxide and *tert*-butyl–OO–*tert*-butyl with this ionization technique in this work confirmed that these compounds resulted in the respective  ion adducts, thus allowing us to distinguish them from each other and from carbonyl products. With C_2_H_5_OO^•^ and i-C_3_H_7_OO^•^, the main ions observed in the presence of metal surfaces were [R_–H_O + NH_4_]^+^, thus indicating that the main products from the surface reactions were the carbonyl compounds acetaldehyde (CH_3_CHO) and acetone (CH_3_C(O)CH_3_), respectively. The next most abundant compounds found to be produced by the surface reactions were the organic peroxides C_2_H_5_OOC_2_H_5_ and C_3_H_7_OOC_3_H_7_, respectively, observed at the ions . Surprisingly, non-negligible signals for these organic peroxides were also observed in the presence of the gas-phase chemistry alone (PFA and glass surfaces), although their formation in the gas phase has not been clearly observed before.^8,42^

With regard to alcohol, a little excess of iso-propanol was observed with metal surfaces, but neither was methanol nor ethanol due to the ineffectiveness of  adduct ionization with these two compounds.

For CH_3_OO^•^, no carbonyl compound (CH_2_O), peroxide (CH_3_OOCH_3_), or even alcohol (CH_3_OH) could be observed by  chemical ionization. This was possibly due to the lower binding energies of the  adducts of these compounds than those of water. Instead, the only significant ion product identified for this radical was *m*/*z* 96.066(10), which was attributed to the sum formula of C_2_H_6_O_3_. Potential isomers of this compound are H_3_C–O–O–CH_2_OH or the organic trioxide (H_3_C–O–O–O–CH_3_) as similar hydrotrioxides (ROOOH) have been reported recently.^43^

## 3 Discussion

The results of this work can be compared with the few data reported in the literature for the losses of ROO^•^ on surfaces. An early study of the uptake of CH_3_OO^•^ and C_2_H_5_OO^•^ by the walls of 1/4" PFA tubing in a PERCA system reported an uptake coefficient of 1.1 × 10^–5^ and 1.2 × 10^–5^ for CH_3_OO^•^ and C_2_H_5_OO^•^ radicals, respectively,^32^ thus somewhat larger than that reported in the present study. A subsequent study of the losses of CH_3_OO^•^ in another PERCA system^33^ reported negligible losses on PFA and borosilicate glass but high losses on metals (stainless steel, gold, and nickel) yet without quantitative measurements, which are nonetheless in better agreement with the present study. Finally, a third study reported the relative losses of CH_3_OO^•^ and HO_2_^•^ on PFA, PTFE, and glass, which cannot be easily converted into absolute loss rates or uptake coefficients.^34^ Comparing the absolute uptake coefficients reported in the present work with those of these previous studies is difficult as the latter were based on indirect measurements of the ROO^•^ by chemical amplification. However, the importance of the uptake of the ROO^•^ on metals compared with that on PFA and glass reported in these previous works is consistent with the observations of the present study.

### 3.1 Proposed Mechanisms

Based on the observed products and variations of the uptake coefficients with the electrochemical potential of the metals, the reactions of peroxy radicals with metals are proposed to proceed by a redox chemical reaction involving electron exchange. The mechanism is likely initiated by the formation of a bond between the radical site and the metal (chemisorption), forming the complex M^*n*^–O–O–R (reaction 1), where M^*n*^ refers to the metal atom oxidation state. M^*n*^–O–O–R can then decompose following different channels (reactions 2a–2c). In the first potential channel, M^*n*^–O–O–R would decompose through the cleavage of the O–O bond, which is the weakest bond in the complex. The metal would thus take up an oxygen anion (O^2–^), and an alkoxy radical would be released in the gas (reaction 2a). In the second potential channel, a hydroxide anion (OH^–^) is taken up by the metal lattice, and a carbonyl compound is directly produced in the gas (reaction 2b). A third possible channel is that the metal takes up both an oxygen and a hydroxide anion to form an oxyhydroxide complex, then releases an alcohol in the gas (reaction 2c).
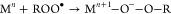
1
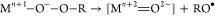
2a
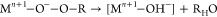
2b
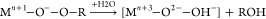
2c

The alkoxy radical produced by channel 2a then reacts quickly, in line with the expected gas-phase oxidation chemistry
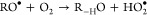
3

It is also possible that either the chemisorbed peroxy groups react between them or that RO^•^ and ROO^•^ present near the surface recombine, which could explain the products observed in this study (reactions 4 and 5).
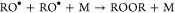
4
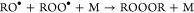
5

Depending on the relative importance of the different channels (2a–2c), these surface reactions could thus either accelerate the conversion of ROO^•^ into RO^•^ compared to the gas-phase chemistry by channel 2a or terminate the radical’s chain reaction with channels 2b and 2c, thus acting as a radical trap.

The mechanism proposed in this work is somewhat different from the one proposed usually in antioxidant chemistry,^3,44^ where the peroxy radical reacts by hydrogen atom transfer (HAT) with another molecule, forming a hydroperoxide (ROOH). The observation of a little ROOH and a large amount of carbonyl compound in the present work suggests that instead of an HAT, the main process under our experimental conditions is the uptake of a hydroxide ion by the metals.

### 3.2 Implications for the Study of ROO^•^

All of the experimental knowledge of organic peroxy radicals and understanding of their reactivity rely on instrumental setups, in which surfaces are invariably present. Our findings demonstrate that reactions on metal surfaces can influence the gas-phase concentration of these radicals and, consequently, the kinetic information derived from them, as observed in chemical amplification.^12^

To mitigate surface reactions in the investigations of the radical gas-phase reactivity, usual strategies are to use glass or Pyrex reactors and/or coat their walls with halocarbon wax or other “anti-sticking” material. For instance, coating a Pyrex glass reactor with boric acid was reported to lead to twice the peroxy radical signal than a stainless steel reactor.^45^ Nonetheless, metals are sometimes the best mechanical choice, particularly to vary the reactor temperature. This has been the reason for designing some simulation chambers in stainless steel or aluminum, like HIRAC,^46^ CESAM,^47^ or AIDA.^48^ But the effects of surface reactions on radical gas-phase concentrations could be especially important in smaller-volume chambers or reactors. For instance, according to the uptake coefficients reported in this work (Table 1, γ = 7 × 10^–5^ for stainless steel), the loss rate of CH_3_OO^•^ on the walls of a stainless steel reactor with an internal radius of 2 cm could reach 0.64 s^–1^, potentially exceeding its gas-phase reaction rates under low radical and NOx concentrations. Similarly, flowing 0.5 sLm of air containing CH_3_OO^•^ through only 10 cm of stainless steel tubing of 1/4″ OD would lead to a 60% loss of the radical concentration. An experimental setup for studying organic peroxy radicals would thus benefit from considering these uptake coefficients to optimize their signal levels and measurements. If not taken into account, such surface losses would lead to an overestimation of the gas-phase reaction rates. They could also impact the product mixtures, thus misleading the analysis of the mechanisms.

## 4 Conclusions

Organic peroxy radicals are key oxidants in many natural and anthropogenic systems, but their reactions with solid surfaces had never been studied until now. In this work, the uptake of organic peroxy radicals by various solid surfaces was studied experimentally thanks to a new technique that allows for monitoring individual radicals directly and in real-time. The radical reactivity was found to vary widely with different surface materials and was especially strong with metals. The measured uptake coefficients followed the electrochemical potential of the metals, thus indicating the occurrence of redox reactions at the surfaces. The time profiles of the uptakes and the products identified in this work indicate that these reactions proceed first by ROO^•^ establishing a bond with the metal, progressively saturating the surface. The complex formed then decomposes; a part is chemisorbed by the solid, while another part is released as a stable product in the gas. Contrary to gas-phase reactions, these surface reactions are driven by the redox potential, leading to distinct mechanisms, efficiencies, and products. The uptake coefficients measured in this work suggest that these surface reactions might impact the detection of organic peroxy radicals and potentially also the knowledge of their gas-phase reactivity.

The new information on these surface reactions reported in this work should also help increase the detection sensitivity of experimental setups toward ROO^•^, minimize secondary chemistry, and improve measurement reliability.

## Supporting Information Available

The raw data that support the findings of this article along with the data processing are openly available in Zenodo at doi.org/10.5281/zenodo.10790197, reference number 10790197. Detailed methods and materials and description of the statistical analysis (PDF)
