# Supplementary material for: Strong Uptake of Gas-Phase Organic Peroxy Radicals (ROO•) by Solid Surfaces Driven by Redox Reactions
Source: JACS Au. 2024 Apr 30;4(5):1875–82. doi: 10.1021/jacsau.4c00060 (PMC11134354; doi:10.1021/jacsau.4c00060)
Supplement: Supplementary file 1 — au4c00060_si_001.pdf [file au4c00060_si_001.pdf]

Supporting Information: Strong uptake of gas-phase organic peroxy radical (ROO˙) by solid surfaces driven by redox reactions

Olivier Durif,*,† Felix Piel,‡ Armin Wisthaler,‡ and Barbara Nozière*,†

†*Department of Chemistry, KTH Royal Institute of Technology, 10044, Stockholm, Sweden*

‡*Department of Chemistry, University of Oslo, 0315, Oslo, Norway*

E-mail: durif@kth.se; noziere@kth.se

1 Experiments Information

1.1 Experimental setup

The experimental setup consisted of a flow reactor, in which the ROO$^•$ were produced photolytically, and connected to tubes made of the different materials of interest, acting as trapping units for the radicals (Fig. S1). The flow reactor was a quartz tube of 3 cm in diameter and 150 cm in length, maintained at atmospheric pressure. Photolysis was performed using 4 UV-C lamps positioned a few centimeters around the quartz tube. The lamps are UV-C Philips TUVT8 - 36 W, emitting almost exclusively at 254 nm. Most of the reactor length was wrapped in aluminum foil to prevent the penetration of UV-C rays, except for an 8 cm irradiation zone toward the end of the flow tube. The entire reactor was enclosed in a chassis equipped with 4 fans to maintain a constant temperature of 293(3) K inside. The

radicals were produced by introducing, in the reactor, their iodinated precursor diluted to concentrations in the range of 0.1 ppm to 2 ppm into the main flow of synthetic air. For this purpose, a flow of a few sccm of nitrogen was bubbled through the pure liquid precursor. Then, the production of $ROO^{\bullet}$ was initiated by the photolysis of the precursor with UV-C lamps, followed by the recombination of the alkyl radical $R^{\bullet}$, produced, with molecular oxygen,

$$RI + h\nu(\lambda = 254\,\mathrm{nm}) \longrightarrow R^{\bullet} + I,$$

$$R^{\bullet} + O_2 + M \longrightarrow ROO^{\bullet} + M$$

The precursors employed in this study were iodomethane (CH_3I), producing methylperoxy radicals ($CH_3OO^{\bullet}$), iodoethane (C_2H_5I) generating ethylperoxy radicals ($C_2H_5OO^{\bullet}$), and iso-iodopropane ($i{-}C_3H_7I$), producing mainly iso-propylperoxy radicals ($i{-}C_3H_7OO^{\bullet}$). The flow of synthetic air into the flow tube was kept constant at 2 sLm, therefore the 8 cm irradiation window corresponded to a gas residence time of approximately 1.5 s, over which a few percent of the precursor was estimated to be photolyzed. This corresponded to a concentration of $ROO^{\bullet}$ between $10^{11}\,\mathrm{cm}^{-3}$ to $10^{12}\,\mathrm{cm}^{-3}$ exiting the flow tube reactor and subsampled in the solid tubes serving as trapping units. To study the uptake of organic peroxy, radicals were flown through tubing made of different materials: aluminum, 316 stainless steel, brass (10 % zink), copper, borosilicate glass, and perfluoroalkoxy alkanes (PFA). These tubes were a few millimeters in diameter (refer to Table S3). The residence time of the radicals on the solid surfaces was controlled by varying either the length of the trapping tubes or the gas flow rate through them. The gas flow through the trapping tubes was varied between 0.34 sLm to 1.2 sLm, by adjusting the mass flow controller, right before the flow entered the spectrometer. Tube lengths were adjusted between 5 cm to 100 cm. This configuration ensured that the pressure in the reactor and the density of the radicals produced remained constant, regardless of the flow through the radical trapping tubes. This design also

allowed the residence time to be limited only by the trapping tube length, with a maximum temporal resolution of the order of a tenth of a second. The radicals and their stable products were monitored using a state-of-the-art PTR-TOF-MS FUSION 10k instrument (IONICON Analytik, Gmbh). Chemical ionization was primarily performed by proton transfer from hydronium ion ($[H_3O]^+$). Additional measurements were also made with ammonium ($[NH_4]^+$) chemical ionization providing additional information on the stable products. Mass spectra were accumulated over 1 s, and data were recorded with iTOF software (IONICON Analytik, Gmbh). The mass resolution of the spectrometer was approximately $m/\Delta m = 7000$, which was sufficient to discriminate most of the relevant isotopic peaks.

The radicals were detected in their protonated form ($[ROO^\bullet + H]^+$). For unambiguous identification, nitric oxide (NO) was periodically introduced into the instrument subsampling flow to suppress all $ROO^\bullet$ radicals. The $ROO^\bullet$ signals were thus obtained by subtracting the residual background (Fig. 1). Once photolysis was initiated, producing the radicals, mass spectra were acquired every second, while the contact time between the radical and the surface was kept constant. The radical signal as a function of experiment time was extracted from these time-dependent spectra (Figs. 2 and S2).

1.2 Potential effect of secondary iodine chemistry

This section discusses the potential impacts of secondary iodine chemistry on the results of this study. First, iodine chemistry could potentially affect the gas-phase reactions, for instance consume the $ROO^\bullet$, which could interfere with the uptake measurements. However, the gas-phase iodine chemistry with organic peroxy radicals is known[S1] to be slow. Thus, adding the documented reactions into kinetic models did not modify the outcome of kinetic analysis. The only exception would be if IO was produced. But this would be limited to the photolytic region of the setup, thus contributing only to the net quantity of $ROO^\bullet$ produced, and would not interfere with the reactions with the surfaces, which occurred in the dark. In addition, some side-chemistry involving iodine would contribute the same way to the

gas-phase radical concentration, regardless of the type of surface exposed. The absence of uptake observed with Teflon surfaces thus suggests that such gas-phase side-chemistry had a negligible impact in our study. Another possibility is that iodine compounds, either the precursors or photooxidation products, stick to the walls and interfere with the measured uptake. Time-dependent monitoring of the iodine precursors in our experiments indicated that they were particularly sticky to the walls. As indicated in the next section, tests were performed in which the tubes were preconditioned by exposing them to the iodinated precursors for various amounts of time before the experiments. These tests showed that, while extended exposure to the precursors somewhat reduced the $ROO^{\bullet}$ uptake, the differences in the results were within the overall uncertainties. Photolysis products from iodine precursors, such as diiodine (I_2), could also stick to the surfaces and interfere with the peroxy radical uptake. By gradually covering the surfaces and acting as a protecting barrier between the radicals and the surfaces, they could lower the $ROO^{\bullet}$ uptake coefficient and account for the observed decrease of the uptake over time observed in the experiments. The uptake coefficients reported in this work would thus underestimate actual ones, and reactions with surfaces would be even more important for $ROO^{\bullet}$ chemistry than reported in this study. However, to minimize such underestimation the uptake coefficients were primarily quantified from the $ROO^{\bullet}$ signal taken after a short exposure time after photolysis.

1.3 Surface conditions

The tubing materials corresponding to the different surfaces studied were used as provided by the manufacturer but preconditioned by flushing dry nitrogen through them for several minutes before each experiment to remove potential volatile pollutants and humidity from the walls. In some cases, the preconditioning also included the exposure of the surfaces to the iodinated precursors for a few minutes to several hours, to check for potential artifacts due to the stickiness of these precursors to the walls. However, this parameter did not affect the results within the reported uncertainty. The results were not affected either when using

repeatedly some surfaces in several experiments. The degree of corrosion of the surface seemed to affect the results, as observed with copper, for which two different surfaces were tested, one recently obtained from the manufacturer and the other highly oxidized after long exposure to outdoor conditions. The uptake coefficients obtained with these two surfaces differed by about 20 %, which is of the order of the uncertainties reported in these results and did not change the conclusion concerning the order between the metallic materials and the galvanic series.

1.4 Uncertainties

The primary sources of experimental uncertainties in this study relate to the state of the surfaces, the stability of the precursor concentration, and the kinetic reaction time. The uncertainty regarding the state of the surface was the most significant factor, leading us to report two coefficients: one measured between 30 s to 60 s after exposure to peroxy radicals, and the other over a longer duration. It's important to note that these exposures, especially the long ones, were influenced by the concentration of peroxy radicals. The uncertainty in the kinetic time primarily arises from the uncertainty in the flow rates and is estimated to be a maximum of 10 %. The uncertainty in the standard deviation of the fit is considered negligible. While the uncertainty in the absolute concentration of peroxy radicals is also significant, the kinetics are in good approximation first-order as shown in the analysis, so this does not constitute a major source of uncertainty in the final results for metals. However, we encountered a challenge as iodoprecursors have a propensity to adhere to the walls of the containment apparatus, resulting in a gradual increase in concentration over several hours. To address this issue and also to provide comparisons across experiments with varying concentrations, we explored various strategies for normalizing the signal. Repetitions and the sensitivity of our instruments let us estimate the overall uncertainty at 20 % for all rate and uptake coefficients reported in this study.

1.5 List of chemicals used

Gases: synthetic air HiQ 5.0 ($\geq$ 99.999 %), N_2 HiQ 5.5 ($\geq$ 99.9995 %), all Linde Gas. Liquids: iodomethane, 99.5 %, stabilized with copper, Alfa Aesar AB; iodoethane, 98 %, stabilized with copper, Alfa Aesar AB ; 2-iodopropane, 97 %, stabilized, Thermo Scientific. The iodoalkanes were placed in glass bubblers and introduced in the reactor by sending controlled flows of N2 through the liquids, followed by a dilution loop. The gas-phase concentration of these compounds in the reactor was determined from the ratio of their flows to the total flow and from their vapor pressure taken at room temperature as, $V_p(CH_3I)$=540 mbar, $V_p(C_3H_7I)$=58 mbar.

1.6 Experiments list

Table S1: $CH_3OO^{\cdot}$ kinetics experiments list.

**Expt. №**	**Material**	**Max time (s)**	**Tube length (cm)**	**Number of pts**	**$[CH_3I]$ (ppb)**
1.1	Aluminum	0.54	5,10,15,20,25	26	354
2.1	Aluminum	0.46	2.5,5,7.5,10,12.5,15,20,25	21	353
3.1	Aluminum	0.46	5,10,15,20	7	354
1.2	Brass	1.4	10,20,30,40	25	354
2.5	Brass	0.9	30	3	353
1.3	Copper	0.7	5,10,20,30	5	354
2.2	Copper	2.0	3,6,10,20,30,50,100	11	353
1.4	Glass	0.65	30	22	354
2.4	Glass	0.55	30	5	353
1.5	PFA	0.65	30	24	354
2.6	PFA	0.55	30	3	353
1.6	Stainless Steel	0.5	2.5,5,7.5,10,15,20	28	354
2.3	Stainless Steel	0.64	30	3	353
3.2	Stainless Steel	0.64	30	3	354

Table S2: $i-C_3H_7OO^•$ kinetics experiments list.

**Expt. №**	**Material**	**Max time (s)**	**Tube length (cm)**	**Number of pts**	**[$i-C_3H_7I$] (ppb)**
1.1	Aluminum	0.7	5,10,15,20,25,30	7	225
13.5	Aluminum	0.5	20	2	225
1.2	Brass	2.8	10,20,30,40,80	6	225
13.6	Brass	0.7	20	3	225
1.3	Copper	0.7,	30	2	225
2.2	Copper	1.0	10,15,20,40	12	516
10.1	Copper	1.2	9.8,152	10	93
11.2	Copper	0.6	10,20,40,60	9	192
12.1	Copper	1.4	10,20	7	188
13.2	Copper	0.5	20	2	225
1.4	Glass	0.7	30	2	225
7.2	Glass	2.4	30	3	695
13.4	Glass	0.7	20	3	225
1.5	PFA	0.7,8.8	30,404	3	225
2.1	PFA	9.3	24,428	7	225
3.1	PFA	14.9	407,684	27	516
5.1	PFA	3.1	10,30,414	11	698
6.1	PFA	17.0	30,414	16	1410
7.1	PFA	24.3	40,414	5	695
11.1	PFA	3.3	7,67	9	192
13.1	PFA	0.4	20	3	225
1.6	Stainless Steel	0.7	5,10,20,30	4	225
2.3	Stainless Steel	0.5	10,15,20	8	516
4.1	Stainless Steel	0.5	30	6	83
7.3	Stainless Steel	2.4	30	4	695
11.3	Stainless Steel	0.6	10,20,30,60	5	192
12.2	Stainless Steel	1.4	10,20	7	188
13.3	Stainless Steel	0.5	20	2	225

2 Statistical Analysis

The data processing from the PTR-TOF-MS was primarily performed using the Julia package MassSpec.jl, specifically developed for our research requirements. This package is provided in Zenodo repository, along with most of the data, allowing to reproduce this work.

The kinetic analysis was performed considering two approaches: a complete kinetic model including gas-phase reactions and surface reactions which was adjusted numerically, and a

simplified kinetic model considering the surface reaction of peroxy radicals and their self-reaction which was solved by the least squares method.

2.1 Analytical solution

In the first approach, an analytical solution was compared with the experimental data by describing the chemical system with the two following reactions:

$$2\,ROO^{\bullet} \xrightarrow{\text{k˙self}} P,$$

$$ROO^{\bullet} \xrightarrow{\text{k'}} P',$$

k_{self} was taken as 3.5×10^{-13} cm^3 s^{-1} for the $CH_3OO^{\bullet}$, and 1.0×10^{-15} cm^3 s^{-1} for the $C_3H_7OO^{\bullet}$, at 298 K according to the IUPAC recommendations.[S2]

The differential equation governing the evolution of this system was thus,

$$\frac{d[ROO^{\bullet}]}{dt} = -2k_{self}[ROO^{\bullet}]^2 - k'[ROO^{\bullet}]. \tag{1}$$

For many of the surfaces studied in this work, $k' >> k_{self}\,[ROO^{\bullet}]$, thus the first term of the right-hand side was negligible and the analytical solution was fitted as:

$$[ROO^{\bullet}](t) = [ROO^{\bullet}]_0 \exp(-k't). \tag{2}$$

This expression was used to model the decays for iso-propylperoxy in the presence of aluminum, stainless steel, copper, and brass, and for methylperoxy in the presence of aluminum, stainless steel, and brass, where the gas-phase reactions were much slower than the surface processes.

In the presence of glass and PFA for both radicals and in the presence of copper for

methylperoxy, when the gas-phase reactions were not negligible, the analytical solution of the differential equation (1) was fitted, as:

$$[\mathrm{ROO}^{\bullet}](t) = \frac{k' \exp(-k't)}{\frac{k'}{[\mathrm{ROO}^{\bullet}]_0} + 2k_{\mathrm{self}}\left(1 - \exp\left(-k't\right)\right)}. \tag{3}$$

In this analysis, the initial concentration of $[\mathrm{ROO}^{\bullet}]_0$ was estimated to be 3.5 % of the initial concentration of precursor, thus of the order of $3.1 \times 10^{11}\,\mathrm{cm}^{-3}$ in the experiments reported in Fig. 2 and S2. This value was estimated from the photon flux emitted by the lamps passing through the flow reactor, the irradiation time, and the photolysis cross-section of the precursor. This value of $[\mathrm{ROO}^{\bullet}]_0$, was finely adjusted to match the kinetic decay of the radicals obtained with PFA, assuming $k' = 0$ (no uptake by surface).

The strength of this method allowed us to reproduce the data by fitting only one parameter. The regressions were solved using the ordinary least squares method.

2.2 Chemical model

In the second step, the reaction rates were confirmed by numerically modeling a reaction network.

For methylperoxy, the following reactions were considered:

$$2\,\mathrm{CH_3OO^{\bullet}} \longrightarrow 2\,\mathrm{CH_3O^{\bullet}} + \mathrm{O_2}, \tag{4a}$$

$$2\,\mathrm{CH_3OO^{\bullet}} \longrightarrow \mathrm{CH_3OH} + \mathrm{HCHO} + \mathrm{O_2}, \tag{4b}$$

$$2\,\mathrm{HOO^{\bullet}} \longrightarrow \mathrm{H_2O_2} + \mathrm{O_2}, \tag{4c}$$

$$\mathrm{CH_3O^{\bullet}} + \mathrm{O_2} \longrightarrow \mathrm{HCHO} + \mathrm{HOO^{\bullet}}, \tag{4d}$$

$$\mathrm{CH_3OO^{\bullet}} + \mathrm{HOO^{\bullet}} \longrightarrow \mathrm{CH_3OOH} + \mathrm{O_2}, \tag{4e}$$

$$\mathrm{HOO^{\bullet}} \longrightarrow \text{uptake}, \tag{4f}$$

$$\mathrm{CH_3OO^{\bullet}} \longrightarrow \text{uptake}. \tag{4g}$$

The rate constants employed were (4a) $2.1 \times 10^{-13}\,\mathrm{cm^{-3}\,s^{-1}}$, (4b) $1.4 \times 10^{-13}\,\mathrm{cm^{-3}\,s^{-1}}$, (4c) $1.6 \times 10^{-12}\,\mathrm{cm^{-3}\,s^{-1}}$, (4d) $2.0 \times 10^{-15}\,\mathrm{cm^{-3}\,s^{-1}}$, (4e) $5.2 \times 10^{-11}\,\mathrm{cm^{-3}\,s^{-1}}$, (4f) $10 \times k'$, (4g) k' as indicated in Table 1.

For iso-peroxypropyl, the model was the following:

$$2\,C_3H_7OO^\bullet \longrightarrow 2\,C_3H_7O^\bullet + O_2, \tag{5a}$$

$$2\,C_3H_7OO^\bullet \longrightarrow C_3H_7OH + C_3H_6O + O_2, \tag{5b}$$

$$2\,HOO^\bullet \longrightarrow H_2O_2 + O_2, \tag{5c}$$

$$C_3H_7O^\bullet + O_2 \longrightarrow C_6H_5O + HOO^\bullet, \tag{5d}$$

$$C_3H_7OO^\bullet + HOO^\bullet \longrightarrow C_3H_7OOH + O_2, \tag{5e}$$

$$HOO^\bullet \longrightarrow \text{uptake}, \tag{5f}$$

$$C_3H_7OO^\bullet \longrightarrow \text{uptake}. \tag{5g}$$

The rate constants employed were (5a) $7 \times 10^{-16}\,\mathrm{cm^{-3}\,s^{-1}}$, (5b) $3 \times 10^{-16}\,\mathrm{cm^{-3}\,s^{-1}}$, (5c) $1.6 \times 10^{-12}\,\mathrm{cm^{-3}\,s^{-1}}$, (5d) $7 \times 10^{-15}\,\mathrm{cm^{-3}\,s^{-1}}$, (5e) $1.5 \times 10^{-11}\,\mathrm{cm^{-3}\,s^{-1}}$, (5f) $10 \times k'$, (5g) k' as indicated in Table 1.

These chemical models were solved using ChemKinLator, a simulator software for chemical networks.[S3]

The first-order rate coefficients of the reactions at the surfaces used in this numerical network validated those obtained with the analytical regression. In particular, they confirmed that for both radicals and in the case of aluminum, stainless steel, brass, and copper (with $C_3H_7OO^\bullet$) the gas-phase reactions were negligible compared to the surface reactions. For $CH_3OO^\bullet$ in the presence of copper and for both radicals in the presence of glass and PFA, the radical self-reaction was non-negligible but other gas-phase reactions were insignificant. Finally, the results showed excellent agreement between the two methods.

As with borosilicate glass and PFA, for which the peroxy radical uptake was negligible compared to the gas-phase reactions, only the upper values of the first order rate coefficients

for the surface reactions, and the corresponding uptake coefficients are reported in Tables 1 and S3. These upper values were obtained considering our uncertainties and by assuming the real concentration of peroxy radicals to be half of the concentration expected by our estimation ($1.5 \times 10^{11}\,\mathrm{cm^{-3}}$ rather than $3.1 \times 10^{11}\,\mathrm{cm^{-3}}$). With this lower radical concentration in the model, the self-reaction was slower (because it is second-order kinetics). Thus, the difference between the model in the gas-phase with $[\mathrm{ROO^\bullet}]_0 = 1.5 \times 10^{11}\,\mathrm{cm^{-3}}$, and the experimental data was assumed to be caused by surface uptake.

Finally, the uptake coefficient, γ, reflecting the fraction of collisions to the surface resulting in radical removal, was obtained from k' using the following equation:[S4]

$$\gamma = \frac{2k'r}{\bar{v}} \tag{6}$$

where r is the tube's inner radius (reported in Table S3), and $\bar{v}$ is the mean molecular velocity. This value is itself determined from the mass of the molecule, m, and the flow temperature, T, as:

$$\bar{v} = \sqrt{\frac{8k_BT}{\pi m}}, \tag{7}$$

where k_B is the Boltzmann constant. Under the experimental conditions, $\bar{v} = 363\,\mathrm{m\,s^{-1}}$ for $\mathrm{CH_3OO^\bullet}$ and $\bar{v} = 287\,\mathrm{m\,s^{-1}}$ for $\mathrm{C_3H_7OO^\bullet}$.

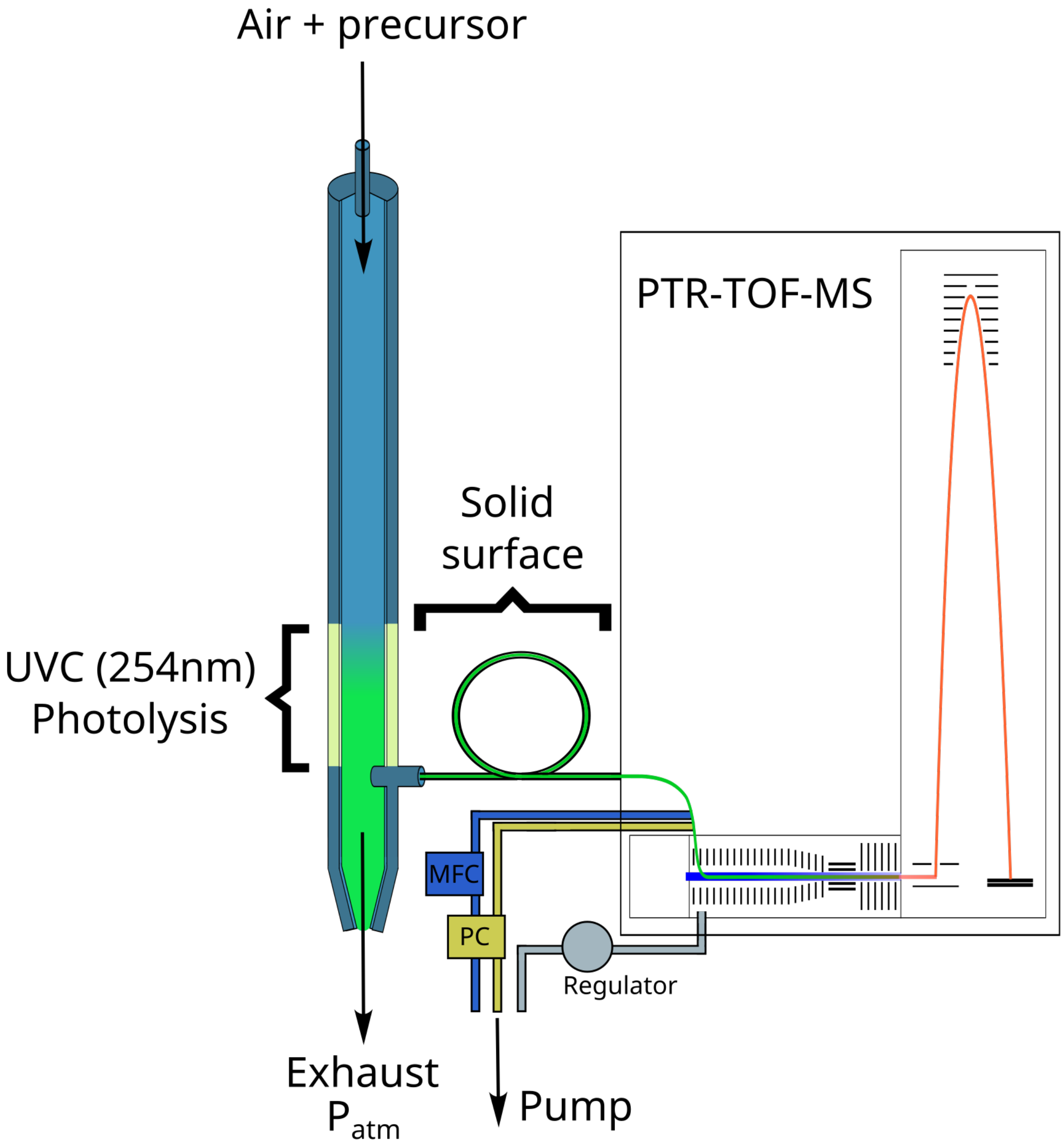

Figure S1: **Schematic of the experimental setup.** MFC: Mass Flow Controler. PC: Pressure Controler.

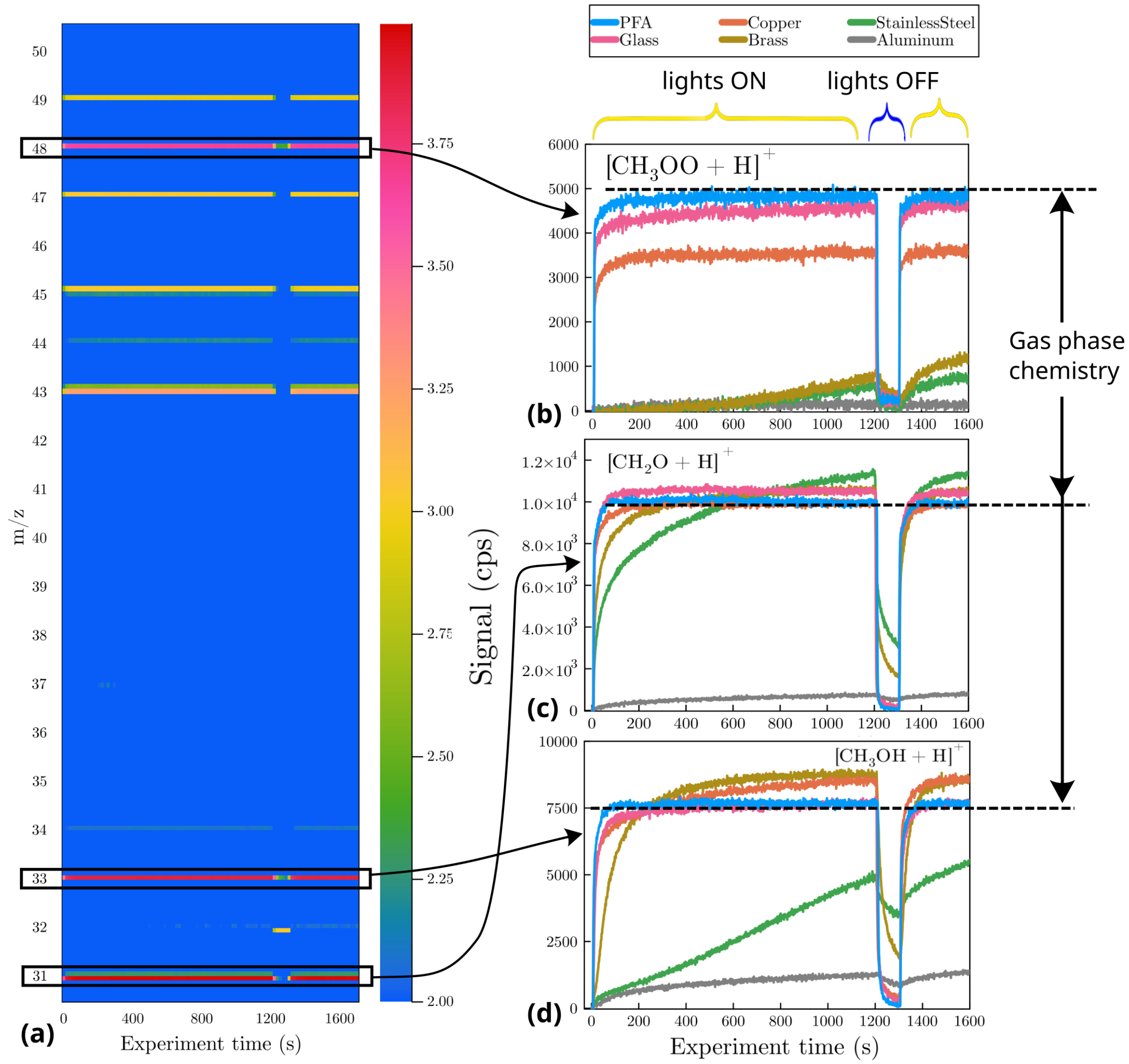

Figure S2: **Time dependent mass spectra, uptake and product formation for $CH_3OO^•$ exposed to various surfaces.** (a) Time evolution of the mass spectra obtained by proton-transfer chemical ionization, after photolysis in the presence of a PFA surface (signal in cps in color code in log scale as a function of ion m/z and experiment time). Time profiles (b) for radical (m/z 48.021, ($[CH_3OO^• + H]^+$)) and main products ions (c) m/z 31.018 ($[CH_2O + H]^+$) (d) m/z 33.033 ($[CH_3OH + H]^+$), with different surface materials.

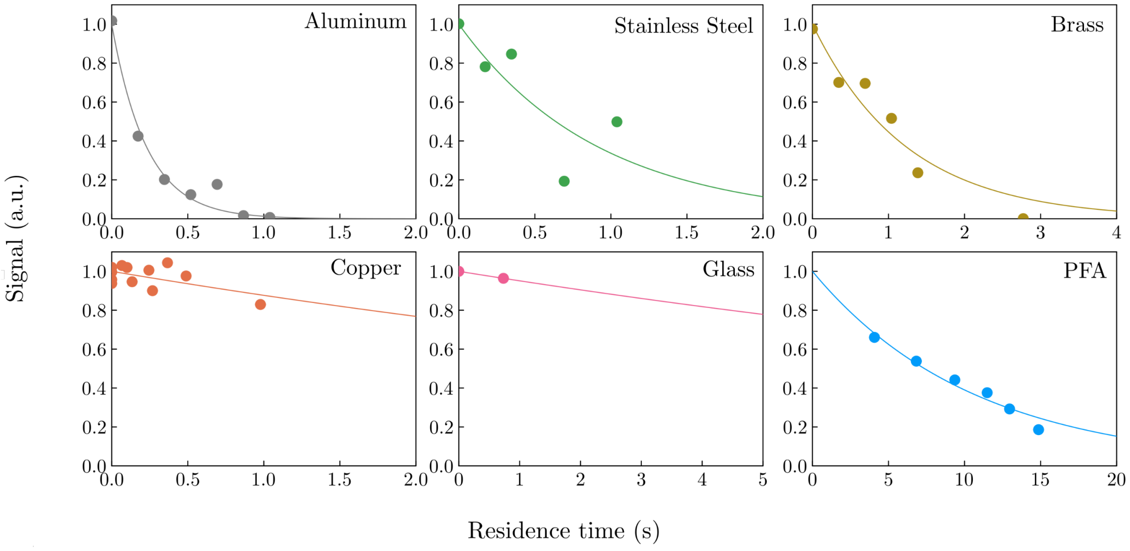

Figure S3: **Decays of $C_3H_7OO^{\bullet}$ as function of residence time with different surface materials.** Similarly to Fig. 3, the radical signal was averaged over an experiment time of 30 s to 60 s following the start of photolysis. These decays were analyzed kinetically to determine the uptake coefficients.

Table S3: **Decay rates and uptake coefficients for $CH_3OO^\bullet$ and i$-C_3H_7OO^\bullet$ by surfaces at long experiment times.** The exposure time was 1600 s for $CH_3OO^\bullet$ and 3000 s for $C_3H_7OO^\bullet$. The uncertainty is estimated to be 20 % and applies both to the rate and uptake coefficients.

		$CH_3OO^\bullet$		$C_3H_7OO^\bullet$	
Material	r (mm)	k' (s^{-1})	γ	k' (s^{-1})	γ
Aluminum	2.0	10	1×10^{-4}	4	6×10^{-5}
Stainless Steel	2.1	2	2×10^{-5}	1	1×10^{-5}
Brass	2.5	0.1	7×10^{-5}	2×10^{-2}	9×10^{-7}
Copper	2.1	$< 5 \times 10^{-2}$	$< 6 \times 10^{-7}$	$< 4 \times 10^{-2}$	6×10^{-7}
Borosilicate glass	2.0	$< 3 \times 10^{-2}$	$< 3 \times 10^{-7}$	$< 1 \times 10^{-2}$	$< 1 \times 10^{-7}$
PFA	1.98	$< 1 \times 10^{-2}$	$< 1 \times 10^{-7}$	$< 5 \times 10^{-3}$	$< 7 \times 10^{-8}$

References

(S1) Dillon, T. J.; Tucceri, M. E.; Crowley, J. N. Rate Coefficients for the Reaction of Iodine Oxide with Methyl Peroxy Radicals. 11, 4011–4018.

(S2) Atkinson, R.; Baulch, D. L.; Cox, R. A.; Crowley, J. N.; Hampson, R. F.; Hynes, R. G.; Jenkin, M. E.; Rossi, M. J.; Troe, J.; Subcommittee, I. Evaluated Kinetic and Photochemical Data for Atmospheric Chemistry: Volume II – Gas Phase Reactions of Organic Species. Atmospheric Chemistry and Physics **2006**, 6, 3625–4055.

(S3) Camacho, E. A. C.; Arguello, J. A. M.; Bastidas, J. A. Á. CHEMical KINetics SimuLATOR (Chemkinlator): A Friendly User Interface for Chemical Kinetics Simulations. Revista Colombiana de Química **2020**, 49, 40–47.

(S4) Shuler, K. E.; Laidler, K. J. The Kinetics of Heterogeneous Atom and Radical Reactions. I. The Recombination of Hydrogen Atoms on Surfaces. The Journal of Chemical Physics **1949**, 17, 1212–1217.
